# A Review on the Occurrence and Analytical Determination of PAHs in Olive Oils

**DOI:** 10.3390/foods10020324

**Published:** 2021-02-03

**Authors:** Valentina Bertoz, Giorgia Purcaro, Chiara Conchione, Sabrina Moret

**Affiliations:** 1 Department of Agri-Food, Environmental and Animal Sciences, University of Udine, 33100 Udine, Italy; bertoz.valentina@spes.uniud.it (V.B.); chiara.conchione@uniud.it (C.C.); sabrina.moret@uniud.it (S.M.); 2 Gembloux Agro-Bio Tech, University of Liège Bât, G1 Chimie des Agro-Biosystèmes, Passage des Déportés 2, 5030 Gembloux, Belgium

**Keywords:** polycyclic aromatic hydrocarbons (PAHs), olive oils, sample preparation, chromatography, occurrence, analytical determination

## Abstract

Polycyclic aromatic hydrocarbons (PAHs) are ubiquitous environmental and processing contaminants, which may contaminate vegetable oils due to atmospheric fall-out or bad production practices. Due to their carcinogenic and toxic effects, surveillance schemes and mitigation strategies are needed to monitor human exposure to PAHs. In particular, due to the lipophilic nature of these substances, edible oils may present unsafe levels of these compounds. Among these, olive oil, and in particular extra virgin olive oil, is a high-value commodity, also known for its health benefits. Therefore, the occurrence of contaminants in this product is not only of health concern but also causes economic and image damage. In this review, an overview of the occurrence of PAHs in all categories of olive oil is provided, as well as a description of the official methods available and the analytical developments in the last 10 years.

## 1. Introduction

Olive oil is a traditional food product that is widely consumed throughout the world, with thousands of years of history, and is an essential component of the Mediterranean diet. Particularly, extra-virgin olive oils (EVOOs) represent a product of high nutritional quality [1], but like all vegetable oils and other foods, may be contaminated with a wide range of lipophilic contaminants, such as polycyclic aromatic hydrocarbons (PAHs).

PAHs are a large class of ubiquitous environmental and processing contaminants produced through incomplete combustion or pyrolysis of organic matter and geological processes [2,3]. They are toxic to various organisms, including humans. Several PAHs have been proven to be genotoxic and mutagenic, whereas other PAHs not defined as carcinogenic can act as synergists [4].

PAHs contain two or more fused aromatic rings and are divided into light PAHs, with up to four fused benzene rings, and heavy PAHs that have up to six benzene rings. Heavy PAHs tend to be more toxic and stable. By virtue of their structure, PAHs have low solubility in water (aqueous solubility decreases for each additional ring added to the PAH) and high solubility in non-polar solvents and edible oils [2].

The presence of PAHs in foods [5,6,7] and vegetable oils [8,9,10,11] has been extensively studied. Hazard and risk characterization is based on the carcinogenic and genotoxic potential of many PAHs. In its assessments of PAHs in food in 2002, the Scientific Committee on Food (SCF) considered the evaluations carried out by various international expert groups and prioritized compounds based on their health risk, rather than their occurrence in food [4]. Survey data show that the most abundant PAHs in food are the low molecular weight PAHs (two or three benzene rings) reported on the Environmental Protection Agency (EPA) list. Such PAHs are less toxicologically relevant and do not contribute to the carcinogenic and genotoxic potential of PAHs. In 2005 the SCF suggested the possibility of using the margin of exposure (MoE) approach [12]. This is similar to the margin of safety (MoS), in which the no-observed-adverse-effect-level (NOAEL) is compared to the human exposure to the xenobiotic. However, this latter approach is not appropriate for carcinogenic and genotoxic substances, for which there is no level without effects. Instead, the MoE is the ratio between a defined point on the dose–response curve for the adverse effects and the human intake. To establish the appropriate point on the dose–response curve the SCF used the benchmark dose (BMD), which is the dose range within which the contaminant is likely to cause a small but measurable tumor incidence. In particular, it has been recommended to use the benchmark dose lower confidence limit 10% (BMDL10), which is the lower limit of the interval of confidence at 95% of the BMD, because this is the lowest effect measurable as statistically significant. According to European Food Safety Authority (EFSA) (2005), for substances that are genotoxic and carcinogenic, a MoE lower than 10,000 is of concern. Based on available dietary intake data from the European population, EFSA (2008) expressed low concern for exposure to PAHs by the average consumer population (MoE > 10,000), while indicating a potential concern (MoE close to or less than 10,000) for high-level consumers and a need for risk management actions.

The effects of PAHs on human health depend mainly on the length and route of exposure, on the amount of PAHs to which one is exposed, and on the innate toxicity of PAHs [13]. Several other factors may also have an impact on health, including subjective factors such as pre-existing health status and age. The ability of PAHs to cause short-term effects on human health is unclear. Occupational exposure to high levels of PAH-containing pollutants causes symptoms such as eye irritation, nausea, vomiting, diarrhoea, and confusion [14]. Mixtures of PAHs are also known to induce skin irritation and inflammation [15]. Health effects from chronic or long-term exposure to PAHs may include decreased immune function, cataracts, kidney and liver damage, respiratory problems, asthma-like symptoms, abnormalities in lung function, and repeated skin contact may induce redness and inflammation [16].

Like many other carcinogenic substances, these hydrocarbons are enzymatically metabolized to various metabolites, some of which are reactive [17]. A serious concern is the ability of their reactive metabolites, such as epoxides and dihydrodiols, to bind to cellular proteins and DNA. The DNA binding of activated PAHs is crucial to the carcinogenic effect. The resulting biochemical disruptions and cell damage can cause mutations, developmental malformations, tumors, and cancer [18,19,20].

The EFSA [21] reported that overall dietary exposure, assuming an individual of 60 kg, is approximately 235 ng/day (for benzo(a)pyrene, BaP), 641 ng/day (PAH2: BaP and chrysene (Ch)), 1168 ng/day (PAH4: BaP, Ch, benz(a)anthracene (BaA) and benzo(b)fluoranthene (BbF)), and 1729 ng/day (PAH8: BaA, Ch, BbF, benzo(k)fluoranthene (BkF), BaP, dibenzo(a,h)anthracene (DBahA), benzo(g,h,i)perylene (BghiP) and indeno(1,2,3-cd)pyrene (IP)). Vegetable fats and oils are a major contributor because of the higher concentration of PAHs in this food group.

### 1.1. European Legislation and Legal Limits

The finding of a highly contaminated pomace olive oil in the Czech Republic in 2001 triggered the process that led to a harmonized European legislation on PAHs.

Before the SCF Opinion in 2002 [4], the 16 EPA priority PAHs [22], (listed in columns 1 and 2 of Table 1), or BaP alone (used as a marker of the presence of other genotoxic carcinogenic PAHs) had been the main focus. The SCF Opinion pinpointed a new list of 15 PAHs, later called the European union (EU)-priority PAHs, demonstrated to be both carcinogenic and genotoxic [4]. In 2005, the member states were required to monitor the 15 EU-priority PAHs together with one additional PAH (benzo(c)fluorene) introduced by the Joint Food and Agriculture Organization (FAO)/World Health Organization (WHO) Expert Committee on Food Additives (JEFCA) [23]. These 16 heavy PAHs are listed in columns 2 and 3 of Table 1.

In the meantime, the EU Regulation n. 208/2005 [24] fixed for the first time a limit for the presence of BaP in different food classes to avoid disparities among the different European member states and in response to food contamination problems as highlighted by the SCF Opinion [4]. After evaluating the data collected from the member states, the EFSA raised doubts on the suitability of BaP as a marker of PAH contamination in food [25]. In 2008 the EFSA published a new opinion suggesting the use of the sum of eight high molecular weight PAHs (PAH8), or a subgroup of four (PAH4), including BaA, Ch, BbF, and BaP [21].

In 2011, Regulation 835/2011 [26] fixed new limits, considering both BaP and PAH4. In particular, for oils and fats, the 2 µg/kg limit for BaP was maintained, whereas a 10 µg/kg limit for the sum of PAH4 was introduced.

No official methods were fixed for PAH determination, but the EU preferred to use the applied methods’ performance criteria. Initially, the method performances were fixed for BaP (Regulation 333/2007 [27]) and were later extended to all the PAH4 (Regulation 836/2011), giving specific guidelines for sampling [28].

### 1.2. Contamination Sources

Contamination of PAHs in vegetable oils is generally explained by the combined effects of various factors and processes, including environmental contamination, oil processing, and migration from food contact materials.

Different authors investigated the impact of environmental contamination on olive oils’ PAH load [11,29,30,31,32,33]. Environmental contamination generally occurs through atmospheric deposition on growing crops. PAHs present in dust and particles from air pollution and smoke can contaminate the olives and the surface contamination can be transferred to the oil during extraction. Therefore, oil is expected to contain background contamination reflecting the environment’s contamination where the olive grows [29].

Contamination of olive fruits occurs on the olive skin and depends directly on the environmental pollution level and inversely on the fruit size. The comparison between manual and mechanical harvesting showed that exposure to diesel exhaust fumes from combine harvesters is the main source of contamination of the olive peel.

High PAH concentrations have been reported in oil from olives harvested in an olive grove in a rural area beside piles of old railway ties [31]. In addition, the practice of firing the field after harvesting is suspected to be a cause of PAH contamination [4].

Contamination with PAHs may also arise from the use of lubricants, extraction solvents, and detergents used during olive oil production. In contrast, washing or talc addition during extraction did not affect PAH levels [29].

In the case of pomace oil, the drying process, by which combustion gases come into direct contact with the olive lees, may lead to high contamination levels. Pomace is typically removed from the mill by a conveyor belt and stored outdoors at the olive mill and/or at the pomace plant. Storage may last up to several weeks. Old and worn out bulldozers, possibly spilling hydraulic oil directly onto the pomace, are used to move the pomace. Moreover, the pomace is exposed to the exhaust of the bulldozers and other vehicles in the lot, as well as to atmospheric fallout [30].

It has also been shown that recycled polyethylene (e.g., plastic bottles) contaminated with PAHs can be an important source of contamination. In fact, PAHs may diffuse back into the vegetable oil and the extent of diffusion depends linearly on the square root of the storage time [34].

A different source of contamination may occur through contact with mineral oil residues (rich in alkylated PAHs). In particular, the practice of storing olives in jute bags treated with mineral oils (before spinning of jute fibers) can cause the migration of PAHs into food [35]. PAH contamination in vegetable oils can also be found when they are transported in tanks previously used for the transport of mineral oils or fuels (such as diesel). As it is difficult to empty completely and adequately clean these tanks between one cargo and the other, this practice should be avoided [36]. The lubricating oil used in installation (conveyor belt) and asphalt debris in the maintenance of extraction plants may be other sources of contamination [30].

The refining process can reduce most contaminants and improve oil quality [28]. The effects of the refining phases on the removal of PAHs from vegetable oils have been studied by many researchers [37,38,39,40,41,42,43,44]. According to these studies, neutralization, bleaching, and deodorization phases provide the most significant reduction in the PAH content. The efficiency of the refining process may depend on the quality of the initial raw material and the initial levels of PAH in unrefined oil, as well as on refining conditions.

### 1.3. Occurrence

There is quite limited information on PAH concentrations in olive oils commonly consumed around the world. Table 2 summarizes the content of PAHs reported in the different categories of olive oil, i.e., EVOO, virgin olive oil (VO), olive oil (OO), and olive pomace oil (OPO). Data were obtained from a literature review of the last decade.

EVOO, which is extracted from olive fruits exclusively by mechanical processes without any further treatment, generally contains very low amounts of PAHs, which can be mainly traced back to environmental sources [45]. As observed in Table 2, in all cases, except for a sample from Syria, PAH4 values were well below the EU legal limits (10 µg/kg). Alomirah et al. [46] found BaP contents that exceeded EU legal limits (2 µg/kg) in only two samples imported from Turkey and Syria. As expected, the level found in VO is on the same order of magnitude as EVOO. In fact, VO does not undergo any additional process compared to EVOO, but it is only characterized by lower sensory quality and higher acidity and oxidation status.

OO, which consists of a blend of refined olive oil and VO, showed higher PAH4 content compared to EVOO. Refining reduces the amount of PAHs, but the extent of reduction depends on the operation conditions and the initial content [47].

Finally, OPO is a low-quality oil produced from exhausted crushed olive lees after drying and subsequent extraction with organic solvents. OPO cannot be used directly for human consumption, undesired minor components and organoleptic properties need to be removed by refining (including degumming, neutralization, partial elimination of waxes, bleaching with activated clays, and deodorization) [48]. In OPO, high PAH levels occur despite the refining process due both to the complexity of the matrix and the high initial content of PAHs [45,49] (Table 2). PAH contamination may occur during pomace storage [50] and the drying and solvent extraction phases [45,49].

## 2. PAH Determination in Vegetable Oils

PAH determination generally consists of three steps: extraction, clean-up or purification, and analytical determination, including detection [62]. Due to the complex nature of the matrices involved, sampling often requires intensive extraction and/or preparation. One of the main difficulties in the analysis of fatty matrices is the high-fat content, requiring laborious and time-consuming procedures [63].

Herein a detailed overview of the official methods available and the most recent (the focus is on the last decade) methods is discussed.

### 2.1. Official Methods

There are several reference methods for PAH determination in vegetable oils, which will be herein described.

*Method ISO 15302* [64] *“Animal and vegetable fats and oils—Determination of benzo[a]pyrene content by reverse-phase high performance liquid chromatography (HPLC)”*, previously approved as an AOAC method in 1997. It is an international standard for the determination of BaP in crude or refined edible oils and fats. The oil sample (approximately 400 mg diluted in 2 mL of solvent) is loaded onto a packed alumina column (22 g) and eluted with light petroleum or hexane (the first 20 mL of eluate are discharged and the next 60 mL are collected). After evaporation to dryness and the addition of benzo(b)chrysene as an internal standard, the residue is dissolved with 1 mL of CH_3_CN/THF (90:10, *v*/*v*) and injected into the HPLC using a fluorometric detector (FLD) set at a BaP-specific wavelength (384–406 nm). The method covers the 0.1–50 μg/kg range.

The method has been reviewed every 5 years and the most recent version [65] shows no changes in the method principle.

*ISO 15753/FDIS* [66] *“Animal and vegetable fats and oils–Determination of polycyclic aromatic hydrocarbons”*. This includes two methods, a general method and a method specific for coconut oil and vegetable oils with short-chain fatty acids, for the determination of 15 PAHs. These ISO methods are not quantitative for the more volatile compounds such as naphthalene (Na), acenaphthene (Ac), and fluorene (F), and neither of them can be used to analyze PAHs in palm oil and olive pomace oil since they fail to remove specific matrix interferences. The quantification limit is 0.2 μg/kg for almost all compounds analyzed (for fluoranthene (Fl) and BghiP the quantification limit is 0.3 μg/kg, and for IP the quantification limit is 1.0 μg/kg). The general method involves a liquid-liquid extraction (LLE) of 2.5 g oil with 3 × 10 mL + 3 × 2 mL CH_3_CN/acetone (60:40, *v*/*v*) followed by a clean-up on a 2-g C18 cartridge, preconditioned with 24 mL MeOH + 24 mL CH_3_CN, and PAH elution with 5 mL CH_3_CN/acetone (60:40, *v*/*v*). An additional purification step is performed on 500 mg Florisil cartridge, preconditioned with 15 mL dichloromethane (DCM) + 12 mL hexane (Hex) and washed with 3 × 1 mL Hex/DCM (75:25, *v*/*v*). This is followed by PAH elution with 4 mL Hex/DCM (75:25, *v*/*v*), concentration and dissolution in CH_3_CN or tetrahydrofuran(THF)/MeOH before injection. Determination is achieved by HPLC-FLD. The updated version, ISO 15753 [66], shows no changes in the method principle.

*ISO 22959* [67] *“Animal and vegetable fats and oils–Determination of polycyclic aromatic hydrocarbons in edible fats and oils by on-line donor–acceptor complex chromatography (DACC) and HPLC with fluorescence detection”* provides a liquid chromatography × liquid chromatography (LC-LC) coupling between a purification column, to selectively retain the PAHs, and the LC column, for the separation. PAHs are electron donors and are therefore able to interact strongly with a stationary phase electron acceptor. The sample is dissolved and injected into the DACC column, which acts as an electron acceptor. This column retains the PAHs (electron donors), isolating them from other matrix components. Subsequently, by changing the solvent’s polarity, PAHs are eluted and introduced (in backflush mode) into the analytical separation column. The individual PAHs are detected at different wavelengths and are identified according to retention time and quantified using external calibration. The limit of quantification (LOQ) for the PAHs is 0.1 μg/kg, with a validated dynamic range from 0.1 μg/kg to 3.5 μg/kg for each individual PAH. Seventeen PAHs can be determined by this method: anthracene (A), phenanthrene (Pa), Fl, pyrene (P), Ch, BaA, BeP, BaP, Per, BghiP, anthanthrene, DBahA, coronene, IP, BaFl BbF, and BkF.

*EN 16619* [68] *“Food analysis–Determination of benzo(a)pyrene, ben(a)anthracene, chrysene and benzo(b)fluoranthene in foodstuffs by gas chromatography mass spectrometry (GC-MS)”*. This European Standard specifies a method for the determination of four of the 16 EU priority PAHs. They are BaA, BaP, BbF, and Ch. The method allows their quantification in the presence of the other 12 EU priority PAHs (BjF, CCP, BkF, DBahA, BcF, DBaeP, BghiP, DahP, DBaiP, DBalP, IP, and 5-MeCh) in extruded wheat flour, smoked fish, dry infant formula, sausage meat, freeze-dried mussels, edible oil (including olive oil), and wheat flour by means of gas-chromatography mass-spectrometry (GC-MS). The extraction of PAHs from solid samples is performed by means of pressurized liquid extraction (PLE). The sample clean-up is performed by means of size exclusion chromatography (SEC), followed by solid phase extraction (SPE). The method has been validated in an interlaboratory study via the analysis of both naturally contaminated and spiked samples, ranging from 0.5 µg/kg to 11.9 µg/kg.

*CEN/TS 16621* [69] *“Food analysis–Determination of benzo(a)pyrene, benz(a)anthracene, chrysene and benzo(b)fluoranthene in foodstuffs by high performance liquid chromatography with fluorescence detection (HPLC-FLD)”*. This Technical Specification specifies a method for the determination of BaP plus BaA, BbF, and Ch in several food matrices. The procedure is based on SEC clean-up, followed by HPLC-FLD and complies with the performance characteristics specified in current legislation. The method can be applied to other matrices, like meat products, fresh fish, paprika, roasted coffee, bread, herbs, breakfast cereals, beer, sunflower oil, olives, and fried tomato, with a LOQ below 0.5 µg/kg. In addition, the method is shown to be applicable also for the quantification of the other 12 PAHs of the 15 + 1 EU priority PAHs set in all matrices listed above and at similar level ranges, except for cyclopenta(c,d)pyrene (CPP), where a UV detection has to be used, obtaining LOQ above 8 µg/kg.

### 2.2. Sample Preparation Method for PAH Determination: Focus on the Last Decade

Table 3 and Table 4 display the analytical methods developed/used for PAH determination in edible oils in the last decade. Table 3 demonstrates methods based on HPLC determination, whereas Table 4 presents methods based on GC determination.

In the sample preparation step, two main approaches have been used, namely direct purification of diluted oil (using SPE, gel permeation chromatography (GPC) or DACC) or a pre-enrichment step (such as saponification or liquid-liquid extraction (LLE) before the subsequent purification step (by means of column chromatography, SPE, or SPME). Two papers have proposed only LLE as a purification step [56,89], and one paper proposed the direct injection of the oil in HPLC-FLD after dilution [73].

#### 2.2.1. Enrichment Step

The enrichment step is used to enhance the sensitivity of the analytical method by removing the bulk of the matrix before a more refined purification of the fraction of interest. This preliminary procedure allows researchers to extend the loading capacity of the purification step, thus leading to an increase in sensitivity. In the case of PAH determination in edible oils, the main components to be preliminarily removed are the triglycerides (TAGs). The most common procedure in this regard is saponification, although it is usually long and solvent-consuming.

Among the papers published recently, only three involve a saponification step, followed by column chromatography [46,72] or SPE [79].

Saponification was performed with 5 M NaOH in MeOH/toluene (2:1, *v*/*v*) at 60 °C for 90 min [72], with 1 M NaOH in MeOH for 30 min [79] or with 2 M KOH in H_2_O/EtOH (9:1, *v*/*v*) [46]. The extract obtained still contained aliphatic and high polar compounds other than PAHs, which could interfere with the analytical determination, requiring a purification step. A lab-packed 8-g silica/alumina column was used by Dost and Ideli [72], followed by HPLC-UV-Vis, whereas Alomirah et al. [46] performed purification through silica column chromatography according to the Grimmer and Boehnke procedure [93] and used GC-MS for the final determination. Akdoğan et al. [79] performed the purification step on a 2-g silica SPE, and PAHs were eluted with 9 mL of toluene. Analytical determination was performed with HPLC-FLD. The method developed by Akdoğan et al. [79] shortened the extraction time, reducing the solvent consumption and lowering the LOD and LOQ due to the higher sensitivity of FLD compared to UV-Vis detection.

An alternative to saponification is LLE, although an additional purification step is usually required to remove the residual TAGs, since their removal is not as efficient as saponification. The most common solvents used have been dimethylformamide (DMF) [54,83,84] and acetonitrile (ACN) alone or mixed with acetone [47,48,55,73,85,86,89,90,92]. The amount of solvent consumed with LLE can vary greatly. Zhou et al. [89], for example, performed LLE with 40 mL ACN saturated with Hex, followed by a wash with 3 mL of hexane for lipid removal. In this case, LLE was used for both extraction and purification of the sample. MS/MS was used to increase the sensitivity by drastically reducing the background without losing specificity for analyte identification.

Except for this latter case, the LLE step was generally followed by an additional clean-up of the extract with SPE, column chromatography, solid-phase microextraction (SPME), or dispersive liquid-liquid microextraction (DLLME). Mohammadi et al. [92] performed an LLE to remove most TAGs before extracting the PAHs by means of microwave-assisted dispersive liquid-liquid microextraction (DLLME) with 100 μL of tetrachloroethylene.

#### 2.2.2. Purification Step

##### Method Based on GPC or Column Chromatography

GPC is often used for removing high molecular weight molecules, such as triglycerides. During the separation procedure, the target compounds experience less loss because there is no chemical interaction between compounds and the column. Wang et al. [80] applied GPC as a non-destructive and automated clean-up method for PAH determination. In particular, purification was performed with the use of glass columns packed with styrene/divinylbenzene beads. PAHs were eluted with a mixture of EtAc/CyHex (1:1, *v*/*v*) at a flow rate of 4.7 mL/min. The fraction collected eluted from 9.5 min to 20 min. Although recovery and repeatability were satisfactory, the sensitivity was rather poor.

##### Methods Based on ISO Standard Methods: DACC Purification

Three methods were published based on the ISO 22959 method [67] that uses a DACC purification column and an LC column for the separation. In particular, Neđeral et al. [74] proposed a simpler configuration compared to the ISO one to avoid the requirement for a dedicated HPLC configuration equipped with a special SPE unit. The authors validated a DACC method in which an HPLC gradient pump was used for sample loading and clean-up, performed on the DACC column connected on-line to the separation HPLC-FLD. A second HPLC pump was used for PAH elution and analysis. Specifically, the sample was loaded into the DACC column and the oil was eluted from the column with isopropanol and sent to the waste. After the oil had been flushed from the purification column, the valve was switched to backflush the column, and PAHs were sent into the separation LC column. The elution was performed with a mixture H_2_O/EtAc/ACN in gradient mode.

**Figure 1:**
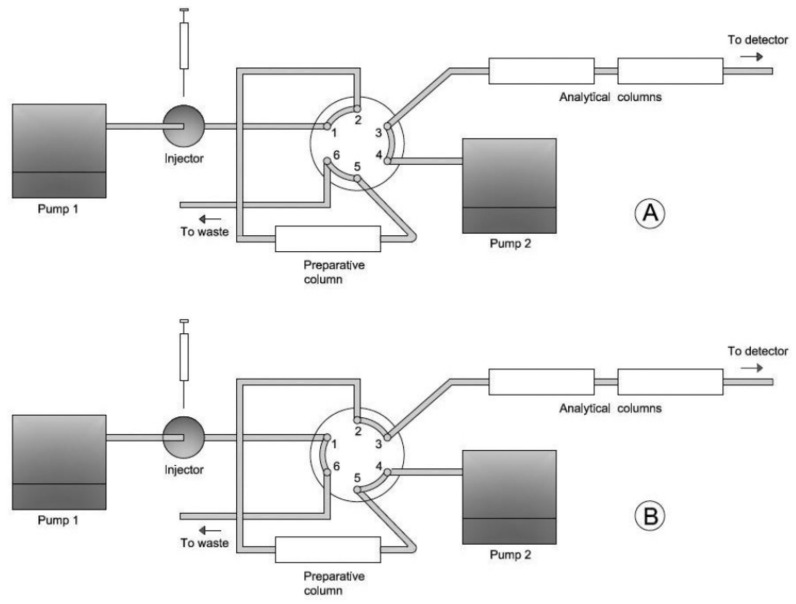
HPLC system used for PAH sampling and determination with loading (**A**) and injecting (**B**) positions of 6-port valve. Reprinted with permission from [74]. Copyright 2015 Copyright HRČAK.

Figure 1 shows a design of the on-line DACC system used by the authors.

Wu and Yu [83] modified ISO method 15753 [66] to reduce the solvent consumption and improve the purification. DMF was used instead of ACN/acetone for the LLE, reducing the presence of interfering peaks derived from triglycerides. For the purification step a Florisil cartridge was used, instead of the coupled use of Florisil + C18, as referred to in the ISO method.

Hollosi et al. [71] performed a DACC column purification followed by LC-MS/MS using a APCI/APPI combination probe as the interface, allowing them to selectively detect the targeted PAHs as well when a difficult matrix such as pomace oil was analyzed.

##### Methods Based on SPE

SPE has been used as a single purification step or after a preliminary LLE or saponification step. In general, most of the methods (19 of 36) performed an SPE as the only sample preparation. Among these, 13 chose ‘classical’ SPE sorbents, including silica [33,84,90], Florisil [83], alumina [61,73,78], dual layer Florisil + zirconia-coated silica/C18 [51], and C18 [54,76,88]. Methods based on MIP-SPE were developed by four authors [52,57,77,86], whereas three chose to perform a magnetic SPE [81,87,91].

##### Classical SPE

**Figure 2:**
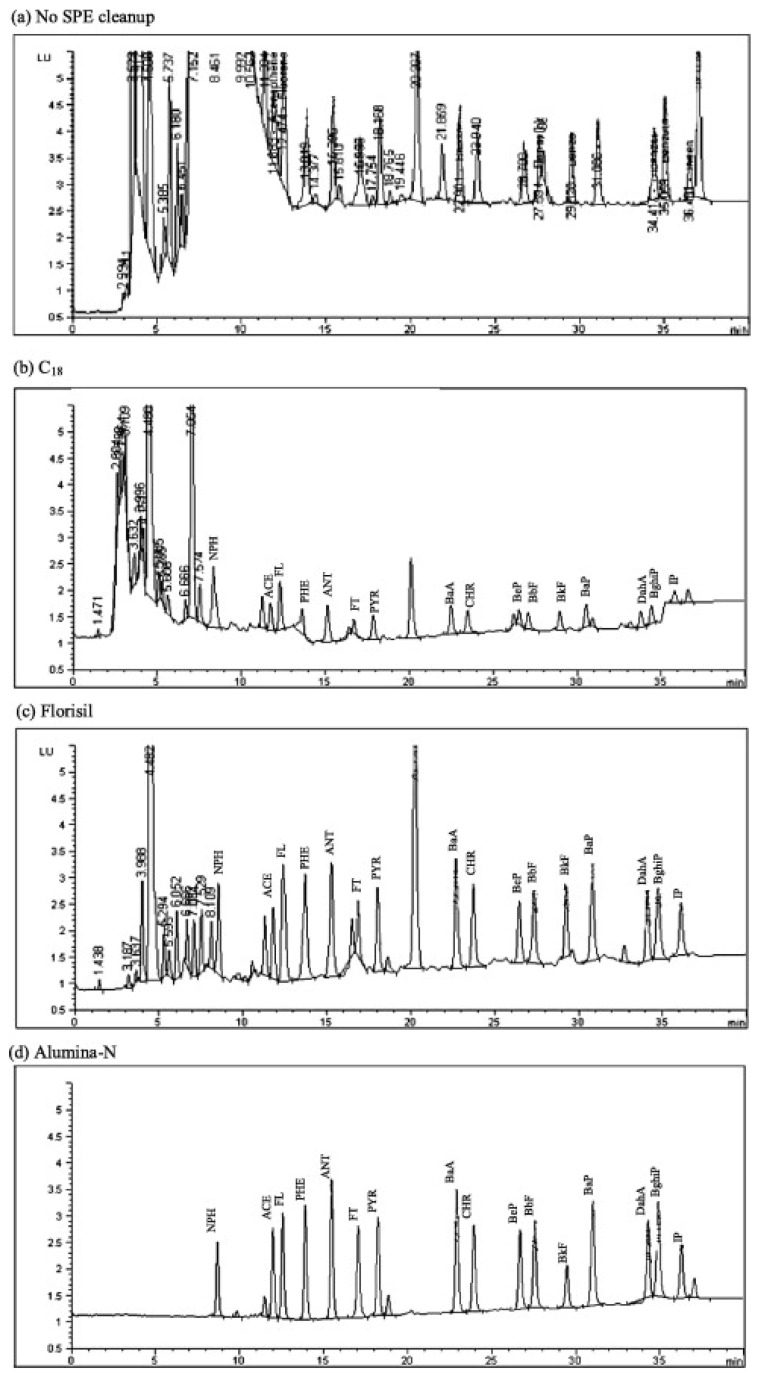
Chromatograms of HPLC-FLD analysis of PAHs after low temperature clean-up followed by (**a**) no SPE clean-up, (**b**) C18, (**c**) Florisil, and (**d**) alumina cartridges [73].

SPE purification can be achieved in two different modes. The first SPE mode makes use of apolar sorbents, which allow researchers to retain the analytes during the sample loading and to elute them with a small amount of organic solvent. The second SPE mode makes use of polar sorbents, retaining the bulk of the matrix (triglycerides) and eluting the targeted analytes. Both cleaning approaches lead to satisfactory results, although a certain degree of sample dependence can be foreseen. Figure 2 compares LC chromatograms obtained without SPE clean-up and with SPE clean-up using different sorbents, namely C18, florisil, and alumina. The latter polar sorbents provided the cleanest chromatogram. Most of the interfering peaks were weakly or highly polar. Recoveries lower than 50% and interfering peaks at the beginning of the chromatogram were obtained after using the C18 cartridge. Prior to SPE, the authors also performed a freezing step in the presence of ACN/acetone (4:1, *v*/*v*), to reduce the fat content of the organic fraction through the precipitation of lipidic compounds [73].

Furthermore, Shi et al. [90] compared different types of SPE cartridges, and RP-C18 in particular showed poor performance, lower intensities of analytes, and dirty chromatograms. Contrarily, alumina, Florisil, and silica cartridges removed most of the interference. The silica cartridge was the best performing in terms of recoveries and removal of interference. As regards silica, these authors managed to reduce volumes of the solvent further, performing sonication and low-temperature fat precipitation before sample purification. In this case, a 500-mg silica cartridge was used and PAH elution was performed with only 4 mL *n*-hexane/DCM (9:1, *v*/*v*).

Rascón et al. [54] proposed a highly sensitive, selective and precise method using a combination of LLE and semi-automated continuous SPE on a 60-mg C18 cartridge, suppressing matrix effects prior to GC-MS detection. The method uses a relatively small amount of organic solvents: 5 mL of *n*-hexane used to dissolve the oil sample, 10 mL DMF/H_2_O (9:1, *v*/*v*) for LLE, and 350 μL ACN for PAH elution from the cartridge. A closed SPE system reduces the risk of sample contamination during manipulation.

##### Molecularly-Imprinted Polymer (MIP)-SPE

In the last decade, commercial solid-phase extraction cartridges based on molecularly-imprinted polymer (MIP) have also been increasingly used. In these methods, a functional monomer and a crosslinker are copolymerized, in the presence of a template molecule. Removal of the template molecule from the obtained polymer by simple solvent extraction makes available the complementary binding sites that can recognize the template molecule from its structurally similar compounds. Multiple interactions (such as hydrogen bonding, ionic, van der Waals, or hydrophobic forces) occur between the PAHs and MIP cavities, which are responsible for their binding.

**Figure 3:**
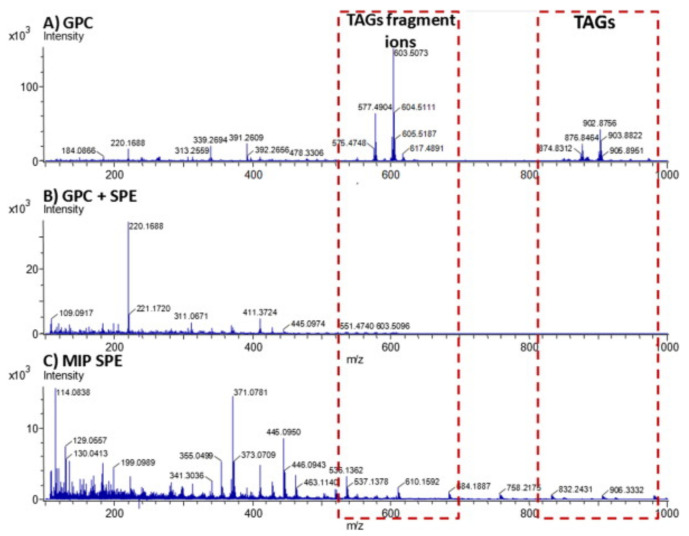
Direct analysis in real time-mass spectrometry (DART-MS) spectral fingerprints (positive ions) after (**A**) GPC-sample purified using GPC, (**B**) GPC + SPE-sample purified using GPC followed by purification using silica mini-column, and (**C**) MIP-SPE-sample extract purified using molecularly imprinted polymer SPE. (TAGs = triacylglycerols) [52].

A rapid detection method of PAHs in edible oils, combining MIP-SPE with GC-MS, was developed by Drabova et al. [52]. According to this method, PAHs were absorbed on 50 mg of the MIP and eluted with 6 mL EtAc. Compared with traditional methods, this method is simpler and easier to perform. Additionally, the procedure was less time consuming, had higher extraction efficiency, and required fewer solvent volumes. Drabova et al. [52] compared the cleaning efficiency of MIP-based sample preparation method with other classical procedures (GPC and GPC followed by SPE on silica) by monitoring the presence of TAGs fragments by direct analysis in real time-mass spectrometry (DART-MS) (Figure 3).

The use of GPC alone failed to reach a sufficient purification level, showing residual lipids (TAGs) in the mass spectrum. To reduce the LOQ and the introduction of a non-volatile matrix into the comprehensive two-dimensional GC-MS system (GC × GC), an additional clean-up step on silica gel SPE was performed. As can be seen in Figure 3, the outcome of the two clean-up steps was comparable with the single step on the MIP-PAH cartridge in terms of TAG purification, although a higher intensity of lower ion fragments can be observed using MIP-SPE. The difference in the interference profiles after the different purification steps was also highlighted in the GC × GC plot. Yu et al. [77] performed MIP purification followed by PAH elution with EtAc, as in the method developed by Drabova et al. [52], however consuming more solvent—10 mL instead of 6 mL. In this case, the analytical determination was performed with HPLC-FLD instead of GC-MS. Similar LOQs were obtained, except for the heaviest PAHs, i.e., IP, DBahA, and BghiP, for which 10-times higher LOQ was obtained by Yu et al. [77]. Another MIP-SPE application was developed by Pschenitza et al. [86], who performed LLE with 3.5 mL ACN before MIP purification. After sample loading into the MIP-SPE cartridge, a wash was performed with 3 mL *n*-hexane, followed by 5 mL of isopropanol. Both the solvents ensure a highly efficient matrix removal, while retaining BaP in the column. PAHs were eluted with 5 mL of DCM and BaP detection was performed through immunoanalytical detection using an anti-BaP enzyme-linked immunosorbent assay (ELISA). Comparing the MIP-SPE results achieved by ELISA quantification and the data acquired from GC-MS (analysis of 15 + 1 EU priority PAHs performed in parallel), it was observed that there was an overestimation of the BaP concentration measured by the ELISA method. Because the anti-BaP antibody used is cross-reactive with other PAHs, the measured ELISA signal has to be considered a sum signal, designated as BaP equivalents. For this reason, the overestimation can be justified as a presumable competition of other PAHs for the interaction with the antibodies used in the ELISA.

Xu et al. [57] used tandem SPE pre-treatment with MIP-PAH and Envi-Carb (graphitized carbon) cartridges to perform simultaneous extraction and purification. The former cartridge was suitable for the determination of heavy PAHs, whereas the latter was suitable for light PAHs. A wash of the solid sorbent was carried out to eliminate matrix components without displacing the analytes. Cyclohexane was chosen as the wash solvent to obtain a satisfactory yield by minimizing co-extracted interferences. Since light PAHs can be easily eluted with toluene from the Envi-Carb cartridge, whereas heavy PAHs require ethyl acetate for an efficient elution, a mixture of toluene/ethyl acetate was selected for PAH elution from the tandem SPE cartridges.

##### Magnetic SPE (M-SPE)

Magnetic nanoparticle (MNP) sorbents used for magnetic-SPE (M-SPE) are nanometer-sized structures with good magnetism and available with various functional groups. After extraction, MNP sorbents with superparamagnetism can be easily separated with the iad of an external magnetic field, facilitating the isolation of the target compounds adsorbed on the MNPs from the sample matrix.

An ideal magnetic sorbent should have strong magnetism to achieve fast magnetic separation, good dispersion properties (to improve the adsorption/desorption kinetics), a large specific surface area to improve the adsorption capacity and extraction efficiency/recovery of target compounds, good selectivity, and good stability. Furthermore, it should be reusable, it should allow for mild adsorption and desorption, and it should be easy to prepare, low cost, and environmentally friendly [94].

M-SPE is an equilibrium-based technique, and under non-equilibrium conditions the amount of analytes extracted depends on the extraction time. A sample preparation method using M-SPE with low-cost carbon nitride (CN) nanosheets was developed by Zheng et al. [87]. CN nanosheets were magnetized and used as sorbents. In this mode, powdery magnetic sorbents can be uniformly dispersed into the sample solution, maximizing the contact between the sorbents and the analytes, thus enhancing the extraction efficiency and facilitating the analyte mass transfer.

The magnetic sorbents were prepared by physical blending and desorption was performed with toluene due to the strong π-π interaction between this solvent and magnetic CN nanosheets. The results demonstrated that the proposed M-SPE was convenient, low-costing, and time-saving. Zhang et al. [91] evaluated the efficiency of a magnetic three-dimensional graphene oxide (GO) nanocomposite. The PAHs adsorbed on 3D-IL@mGO were isolated under a magnetic field. The 3D-IL@mGO material was then washed with acetone to remove the lipids adsorbed, which affected the detection limits. The target PAHs were finally desorbed with toluene (0.5 mL) with the aid of ultrasonic agitation. The ultra-large specific surface area of the 3D, interconnected structure of the adsorbent guarantees a very short extraction time. No significant difference between the M-SPE method and the MIP-SPE method was observed, except for light PAHs (two or three aromatic rings). Furthermore, the solvent consumption, cost, and analysis time required for M-SPE methods were generally lower than those for the MIP-SPE methods.

##### Methods Based on SPME

**Figure 4:**
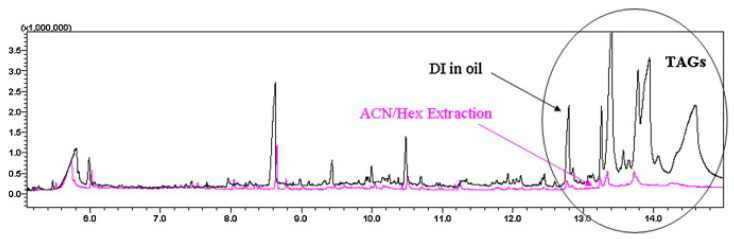
Comparison between the total ion chromatograms (TIC) obtained by direct immersion (DI) of the fiber in the oil/hexane solution, as reported by Purcaro and co-workers [95], and by the DI in hexane after ACN extraction of the oil sample as described in the text [85].

Solid-phase microextraction (SPME) is a widely diffused technique to minimize solvent consumption. Purcaro et al. developed two rapid methods based on direct immersion SPME for the determination of PAHs [95] and BaP [96] in vegetable oils diluted in hexane (200 μL of oil dissolved in 1.3 mL of hexane), reaching the method performances required by the present legislation. In 2013, the same authors [85] improved method sensitivity and robustness by introducing a preliminary low-volume LLE step with 3 mL of ACN to reduce the amount of co-extracted TAGs as much as possible, and back-extracting the PAHs with 4 mL of hexane before deepening the Carbopack Z/PDMS fiber in the sample extract. The coating phase used is highly specific for establishing π–π interactions that allowed a selective extraction of PAHs from the oil matrix. The pre-purification step allowed the authors to obtain results lower than the direct immersion in the oil solution or comparable results in a shorter time (10 min rather than 30 min), but with a significant reduction in the injection of TAGs, which affects the robustness of the method and the fiber and column life (Figure 4).

### 2.3. Analytical Determination

Concerning the analytical determination, HPLC coupled to a fluorescence detector (FLD) has been widely used for the determination of PAHs in food. Liquid chromatography, in combination with ultraviolet/visible (UV-VIS) and diode-array detection (DAD), was also used for the determination of PAHs. However, FLD is considered the first-choice option because of its superior specificity and sensitivity [72]. LC-MS, widely used in food contaminants analysis due to the higher selectivity and sensitivity that it may provide, still has a very limited use in this specific application, as also reported by [97] and even more in the specific field of olive oil PAH determination [56,71]. Most probably, this is due to the difficulty in obtaining an efficient ionization, which requires the addition of dopants, thus complicating the overall procedure [6].

On the contrary, MS is the detector of choice coupled to GC due to its high selectivity and sensitivity. The most critical aspect in the development of a GC method is the selection of an appropriate stationary phase for the separation of critical PA isomers (e.g., A and Pa, Ch and triphenylene, CPP and BaA and Ch, BbF and BjF and BkF, DBahP and DBacA, and DBahA and IP). Medium polar stationary phases are generally preferred to maximize separation by thoughtful optimization [85].

A critical point in the GC analysis of PAHs is related to the discrimination observed for high molecular weight compounds. This discrimination is usually caused by a careless selection of the injection mode and the characteristic of the GC inlet. Multi-baffle and packed liners reduce the discrimination related to an unequal transfer of the analytes from the inlet into the column. Nevertheless, the choice of a suitable packed material is fundamental since active sites or the activation of the material (as is often the case with glass wool) can lead to losses due to too strong retention of the target analytes or to peak distortion [6]. Injection techniques such as temperature-programmed vaporization (PTV) and on-column injection are often applied to reduce discrimination at the injector port [98]. A decrease in the response intensity of high boiling components may also be due to strong interaction with the stationary phase, resulting in broad peaks.

Quadrupole electron ionisation (EI) MS is the most employed detector. Differently from LC-FLD, it allows the detection of all PAHs, including alkylated and non-fluorescence ones. PAHs are highly stable molecules that produce few fragments in EI mode (mainly the molecular ion); thus, the exploration of other ion sources is not worth the effort. Usually, GC-MS is operated in selective ion monitoring (SIM) mode with the advantage of lowering the detection limits [99]. Tandem MS (MS/MS) has been used to enhance sensitivity and specificity, solving co-elution problems with matrix components, mainly of heavy PAHs [89,100].

It is important to highlight that the use of MS allowed the use of isotope-labeled internal standards, which provide a higher accuracy in the overall determination. Indeed, they can be added at the beginning of the entire procedure and they mimic perfectly the behavior of the target analytes at each sample preparation step until the final determination [101].

#### Use of Coupled Chromatographic Techniques

Sample preparation for PAH analysis, if carried out with traditional procedures, is inadequate for the rapid and environmentally friendly determination of PAHs in edible fats and oils. Usually, large solvent volumes are involved, thus requiring a very high concentration factor to achieve a reasonably low detection limit. Of course, this operation results in a concentration of potentially interfering impurities and the loss of the most volatile PAHs. These disadvantages can be overcome by performing sample preparation with a simple LC step, aiming at separating PAHs from TAGs and other interferants prior to their analytical determination. To combine both advantages of GC and LC, on-line hyphenated techniques (LC-GC or LC-LC) could reduce the amount of solvent, sample manipulation, and detection time [35]. More dedicated PAH analytical columns and databases are expected to make the analysis process more accurate and effective. Multidimensional analytical techniques, such as comprehensive GC (GC × GC), can also be considered from a similar viewpoint. In fact, the increased separation power obtained by coupling two columns with different selectivity can enable the isolation of matrix interferences; thus, in several cases, a less intensive clean-up step can be performed [52,95].

The application of LC-GC is also worthy of mention, although no new methods have been proposed recently. This technique is today widely used for the determination of alkylated PAHs from mineral oil contamination, but in the past, it has been optimized for the determination of parent PAHs as well [6,7].

**Figure 5:**
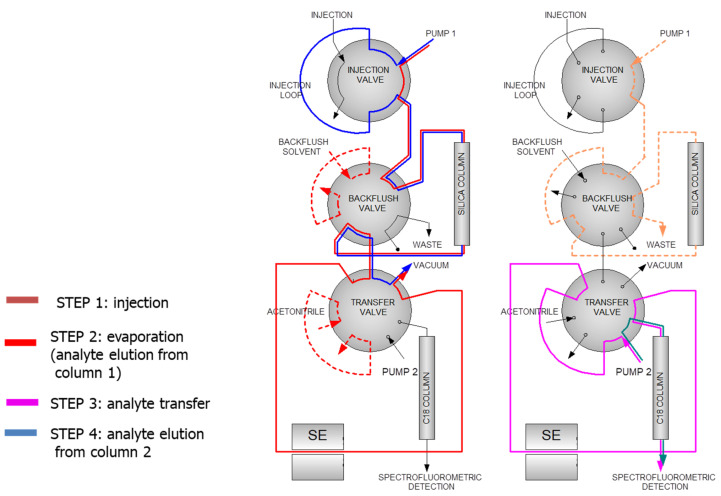
Valve configuration for on-line RPLC-LC determination of heavy PAHs.

The first application of on-line LC-GC for PAH determination in vegetable oils dates back to 1991 [102]. The authors used a silica column to isolate the PAH fraction, which was then transferred to the GC-MS. Moret et al. [103] developed a method to analyze PAHs in oil and lipid extract using a normal phase (NP) silica column coupled on-line with a reversed-phase (RP)-HPLC (C18 column) and an FLD detector. An on-line packed solvent evaporation chamber connected to a vacuum source allowed researchers to evaporate the solvent while trapping the analytes. The analytes were then eluted onto the second column using acetonitrile, whereas the first column was rinsed in backflush mode to eliminate the triglycerides. Figure 5 shows the complex valve configuration used for this application.

An LC-LC method developed by researchers from Unilever has been approved as an ISO method (ISO/FDIS 22959:2009) [67] as described in Section 2.1, “Official Methods”.

In recent decades, comprehensive two-dimensional gas chromatography (GC × GC) has shown an exponential growth in applications. However, the use of such a powerful technique for the determination of PAHs in olive oil is limited to two studies focused on the determination of the 15 + 1 EU PAHs [52,95]. Both methods used a combination of mid-polar and apolar columns, but with two opposite configurations, obtaining equally good separation and satisfactory sensitivity, due also to the separation of the target analytes from matrix interferences thanks to the enhanced separation power obtained by GC × GC.

## 3. Conclusions

PAHs are an important class of food contaminants with a high occurrence in edible oils, including olive oil. The reported data show that the commercial category of olive oil relates somehow to the level of PAH contamination; thus, for instance, pomace oil, the lowest quality category, showed the highest level of contamination. As safety is an important aspect of food quality, this has led to the necessity of the development of rapid and efficient analytical methods. In this regard, although many innovative analytical approaches have been proposed for the analysis of these contaminants in food, it is surprising that, despite the high economic value and health impact of olive oil, the methods remain tied to more traditional and sometimes time-consuming approaches. The use of more environmentally friendly and miniaturized methods has been proposed, such as MIP-SPE, M-SPE, SPME, and DLLME, but it is still limited. Moreover, the determination of critical matrices for pomace olive oil still needs substantial improvement.

Therefore, it is desirable that more rapid, powerful, and innovative approaches enter the field of olive oil, enhancing consumer confidence in the quality and safety of such an important commodity.

## Figures and Tables

**Table 1:** Names and abbreviations of Environmental Protection Agency (EPA)- and EU- priority polycyclic aromatic hydrocarbons (PAHs).

EPA-Priority PAHs		EPA- and EU-Priority PAHs		EU-Priority PAHs + Benzo(c)fluorene	
Compound	Abbreviation (Ring Number)	Compound	Abbreviation (Ring Number)	Compound	Abbreviation (Ring Number)
Naphthalene	Na (2)	Benzo(a)anthracene	BaA (4)	Cyclopenta(c,d)pyrene	CPP (5)
Acenaphthene	Ac (3)	Chrysene	Ch (4)	Benzo(e)pyrene	BeP (5)
Acenaphthylene	Acy (3)	Benzo(b)fluoranthene	BbF (4)	5-Methylchrysene	5-MeCh (4)
Fluorene	F (3)	Benzo(k)fluoranthene	BkF (4)	Benzo(j)fluoranthene	BjF (5)
Phenanthrene	Pa (3)	Benzo(a)pyrene	BaP (5)	Dibenzo(a,l)pyrene	DBalP (6)
Anthracene	A (3)	Dibenzo(a,h)anthracene	DBahA (5)	Dibenzo(a,e)pyrene	DBaeP (6)
Fluoranthene	Fl (4)	Benzo(g,h,i)perylene	BghiP (6)	Dibenzo(a,i)pyrene	DBaiP (6)
Pyrene	P (4)	Indeno(1,2,3-cd)pyrene	IP (5)	Benzo(c)fluorene	BcF (4)

**Table 2:** PAH contents (µg/kg) in extra virgin olive oil, virgin olive oil, olive oil, and olive pomace oil.

	Country	n	EPA 16 PAHs Min–Max (Mean)	PAH8 Min–Max (Mean)	PAH4 Min–Max (Mean)	BaP Min–Max (Mean)	References
Extra virgin olive oil	TR, SY, IT, ES, PS, TN, LB, CA, SA	21	1.09–182.22 (37.88)	0.15–9.62 (1.81)	ND	0.06–6.77 (0.53)	[46]
	TR, ES	9	23.25–62.55 (31.54)	9.45–25.02 (16.04)	0.82–1.37 (1.02)	0.08–0.54 (0.27)	[47]
	ES, IT, TR, TN, GR, MA, USA	4	9.9–48.3 (22.5)	ND	ND	<LOD	[51]
	GR, ES, IT	10	NS	NS	1.99–21.2 (3.85)	0.2–3.75 (0.84)	[52]
	SY	9	33.4–82.4 (54.8)	4.37–36.4 (19.2)	0.34–20.2 (7.66)	NS	[53]
	TN	5	11.4–45.8 (33.7)	0.4–1.0 (0.8)	0.2–0.6 (0.5)	tr	[33]
	MA, IT, ES	8	0.07–4.32 (2.25)	ND	0.04–0.51 (0.35)	0.018–0.075 (0.036)	[54]
	KR	1	ND	ND	2.508	0.481	[55]
Virgin olive oil	TR, SY, IT, PS	7	7.43–124.07 (55.01)	0.41–7.86 (1.88)	ND	0–6.31 (0.90)	[46]
	TR	1	126.97	75.02	0.65	0.36	[47]
	SY, TN, TR	10	39.4–96.7 (63.7)	8.88–46.4 (26.6)	1.48–19.8 (9.29)	0.26–6.71 (1.87)	[53]
	ES	2	4.42–6.36 (5.4)	0.27–0.42 (0.14)	0.15–0.32 (0.24)	0.045–0.058 (0.051)	[54]
Olive oil	SY, TR, JO, IT, ES	7	3.63–192.54 (58.91)	0.26–8.98 (2.09)	ND	ND	[46]
	TR	3	23.48–42.18 (30.67)	10.31–20.08 (13.74)	0.50–0.99 (0.80)	0.24–0.26 (0.25)	[47]
	CN	3	24.11–43.78 (31.85)	ND	1.82–3.46 (2.84)	0.26–0.62 (0.41)	[56]
	CN	10	NS	5.94–10.02 (7.37)	ND–8.17 (4.54)	0.48–0.80 (0.50)	[57]
	Not specified	5	NS (19.05)	NS (1.90)	NS (1.28)	<LOQ	[58]
	CN	8	17.90–55.55 (38.17)	ND	1.48–4.67 (2.88)	0.27–0.89 (0.46)	[59]
	KR	53	ND	ND	0.42–4.07 (2.05)	ND–1.15 (0.22)	[60]
Olive pomace oil	ES	6	28.96–86.03 (48.20)	1.19–6.44 (4.35)	ND	0–3.62 (2.06)	[46]
	TR, ES	6	28.29–179.8 (80.91)	13.04–112.5 (51.9)	0.75–15.08 (4.20)	0.18–3.64 (0.96)	[47]
	NS	5	NS (28.37)	NS (3.90)	NS (3.32)	NS (0.30)	[58]
	PL	3	ND	ND	1.11–3.15 (2.13)	ND–0.25 (NS)	[61]
	IT, ES	3	1.21–2.85 (2.21)	1.13–2.53 (1.73)	0.99–1.97 (1.39)	0.04–0.15 (0.092)	[54]

n: number of samples; ND: not detected; NS: not specified; LOD: limit of detection; LOQ: limit of quantification; tr: trace (<0.05 µg/kg). TR, Turkey; SY, Syria; IT, Italy; ES, Spain; PS, Palestine; TN, Tunisia; LB, Lebanon; CA, Canada; SA, Saudi Arabia; GR, Greece; MA, Morocco; USA, United States of America. JO, Jordan; CN, China; KR, Korea.PL, Poland.

**Table 3:** HPLC methods used for PAH determination in olive oils (2010–2020).

Target Analytes	Sample Preparation	Chromatographic Conditions	Detection	Performance Characteristics	Ref.
15 EU priority PAHs + A, P, Fl	According to ISO 15753:2016 (modified in-house)	Vydac 201TP54 C18 column, 250 × 4.6 mm (5 µm)—Mobile phase ACN/H_2_O in gradient mode	FLD/DAD (222 nm)	Recoveries: 30.3–120.7%; RSD: 0.52–14.68%; LOD: 0.005–2.155 µg/kg	[70]
15 of 16 EPA priority PAHs	Around 1 mL oil diluted in IPA—sample purification via DACC (ChromSpher PI DACC column; mobile phase EtAc/ACN at 2.5 mL/min)	ChromSpher C18 column, 250 × 2.1 mm (5 µm)—Mobile phase: MeOH/H_2_O/EtAc in gradient mode	DA-APPI-MS/MS	Recoveries: NS; RSD: <5%; LOD: 0.19–0.36 µg/kg	[71]
9 PAHs	50 mL oil-saponification (60 °C × 90′) with 1 M NaOH in MeOH/toluene 2:1 (*v*/*v*)‚ LLE with 20 mL toluene-concentration-column chromatography on 8 g silica/alumina (1:1) preconditioned with 20 mL Hex, wash with 30 mL Hex, elution with 40 mL DCM/Hex 80:20 (*v*/*v*)—concentration and dissolution in ACN	Spherisorb C18 column, 250 × 4.6 mm (5 µm)—Mobile phase: ACN/H_2_O (isocratic)	UV–Vis	Recoveries: NS; RSD: 0.35–1.60%; LOD: 0.26–1.15 µg/kg; LOQ: 0.87–3.84 µg/kg	[72]
15 of 16 EPA priority PAHs	2.5 g oil-LLE with 10 mL acetone/ACN 40:60 (*v*/*v*)—sonication-SPE on C18, preconditioned with ACN/MeOH, elution with 5 mL acetone/ACN-Florisil preconditioned with DCM + *n*-Hex, wash with 2 × 1 mL Hex/DCM 75:25 (*v*/*v*), elution with 4 mL *n*-Hex/DCM 75:25, (*v*/*v*)—concentration and dissolution in THF/MeOH	ZORBAX Eclipse PAH C18 column, 4.6 × 250 mm (5 µm)—Mobile phase: ACN/H_2_O in gradient mode	FLD	Recoveries: NS; RSD: NS; LOD: NS; LOQ: NS	[47]
16 EPA priority PAHs	1 g oil-low temperature fat precipitation with ACN/acetone 4:1 (*v*/*v*)-SPE on alumina, elution with 10 mL Hex/DCM 1:1 (*v*/*v*)—concentration and dissolution in ACN (tested also Florisil and C18 cartridges)	PAH-C18 column, 250 × 4.6 mm (5 µm)—Mobile phase: ACN/H_2_O in gradient mode	FLD	Recoveries: 62–118%; RSD: NS; LOD: 0.13–3.13 µg/kg; LOQ: 0.25–6.25 µg/kg	[73]
16 EPA priority PAHs	4 g oil in cyclohexane-After sample loading, DACC column washed with IPA, PAH elution (backflush) with H_2_O/EtAc/ACN in gradient mode (direct coupling with the HPLC column)	Pursuit 5 PAH C18 column, 250 × 4.6 mm (5 µm)—Mobile phase: H_2_O/EtAc/ACN in gradient mode	FLD	Recoveries: 94.1–109.4%; RSD: 2.3–4.7%; LOD: 0.04–0.61 µg/kg; LOQ: 0.13–1.84 µg/kg	[74]
12 of 16 EPA priority PAHs	0.5 g oil in 1 mL THF-Dilution with 2 mL THF/ACN (1:1, *v*/*v*)—direct injection	Supelcosil^TM^ LC-PAH RP-C18 column, 250 × 4.6 mm (5 µm)—Mobile phase: ACN/H_2_O in gradient mode	FLD	Recoveries: 87.6–98.7%; RSD: intra-day 0.75–8.96% inter-day 1.93–11.59%; LOD: 0.07–0.61 µg/kg; LOQ: 0.23–2.04 µg/kg	[75]
16 EPA priority PAHs	1 g oil-LLE with 10 mL ACN/Acetone 60:40 (*v*/*v*)—sonication—SPE on C18, preconditioned with 20 mL ACN, elution with ACN/Acetone (60:40, *v*/*v*)—concentration and dissolution in ACN	Supelcosil LC-PAH C18 column, 250 × 4.6 mm (5 µm)—Mobile phase ACN/H_2_O in gradient mode	FLD/DAD (228 nm)	Recoveries: 59.5–94.6%; RSD: 0.48–4.98%; LOD: 0.01–2.35 µg/kg	[76]
12 of 16 EPA priority PAHs	0.5 g oil in petroleum ether—MIP—SPE (0.5 g) preconditioned with 5 + 2 mL of petroleum ether—washinf with 5 mL of petroleum ether, PAH elution with 10 mL of EtAc—concentration and dissolution in ACN	Supelcosil LC-PAH C18 column, 250 × 4.6 mm (5 μm)—Mobile phase: ACN/H_2_O in gradient mode	FLD	Recoveries: 56.0–109.1%; RSD: 2.2–7.1%; LOD: 0.04–0.66 µg/kg; LOQ: 0.14–2.25 µg/kg	[77]
15 of 16 EPA priority PAHs	1 g oil—low temperature fat precipitation with 8 mL ACN/acetone 4:1 (*v*/*v*)—SPE on alumina, elution with 10 mL Hex/DCM 1:1 (*v*/*v*)—concentration—SPE on amino phase, preconditioned with 30 mL Hex, elution with 25 mL Hex/toluene 70:30 (*v*/*v*)—concentration and dissolution in ACN	Zorbax Eclipse PAH C18 column 150 × 4.6 mm (5 μm)—Mobile phase: ACN/H_2_O (isocratic)	FLD	Recoveries: 81–114%; RSD: 3–10%; LOD: 0.09–1.97 µg/kg; LOQ: 0.29–5.99 µg/kg	[78]
16 EPA priority PAHs	1 g oil-LLE (2 × 8 mL ACN)—filtration—concentration	Zorbax Eclipse PAH C18 column, 100 × 2.1 mm (3.5 µm)—Mobile phase: ACN/H_2_O in gradient mode	APPI-MS/MS	Recoveries: 77.8–106.4%; RSD: 2–7.5% (intraday), 2.5–8.9% (interday), LOD: 0.006–0.156 µg/kg	[56]
15 of 16 EPA priority PAHs	0.4 g of oil SPE: C18 (bottom)/Florisil (upper layer), preconditioned with 10 mL acetone, elution with 15 mL ACN—concentration	Supelcosil LC-PAH C18 column, 250 × 4.6 mm (5 µm)—Mobile phase ACN/H_2_O in gradient mode	FLD	Recoveries: 79–123%; RSD: 5–16% (intraday), 10–68% (interday); LOD: 0.19–1.01 µg/kg	[51]
15 of 16 EPA priority PAHs	2.5 g oil-UASE with 10 mL acetone/ACN 40:60 (*v*/*v*)—SPE on C18, preconditioned with 24 mL ACN/MeOH, elution with 5 mL acetone/can—Florisil preconditioned with 15 mL DCM + 12 mL Hex, elution with 9 mL Hex/DCM 3:1 (*v*/*v*)—amino phase, preconditioned with 30 mL hexane, elution with 25 mL Hex/toluene 70:30 (*v*/*v*)—xoncentration and dissolution in ACN	Zorbax Eclipse C18 column 150 × 4.6 mm (5 μm)—Mobile phase: ACN/H_2_O (isocratic)	FLD	Recoveries: 75–110%; RSD: 3–8%; LOD: 0.19–0.97 µg/kg; LOQ: 0.57–2.93 µg/kg	[48]
PAH4	1 g oil—saponification (60 °C × 30′) with 10 mL 5 M methanolic KOH—LLE with 15 mL toluene—SPE on 2 g silica preconditioned with 8 mL toluene, elution with 9 mL toluene—concentration and dissolution in ACN	Eclipse Plus C18 column, 50 × 4.6 mm (1.8 μm)—Mobile phase ACN/H_2_O/EtAc in gradient mode	FLD	Recoveries: 78.2–87.2%; RSD: 3.25–6.81%; LOD: 0.06–0.12 µg/kg; LOQ: 0.13–0.24 µg/kg	[79]
16 EPA priority PAHs	0.4 g oil in 10 mL cyclohexane—GPC on glass column packed with styrene divinylbenzene beads, elution with EtAc/CyHex 1:1 (*v*/*v*)—concentration and dissolution in ACN	BEH Shield RP-C18 column, 150 × 2.1 mm, (1.7 μm)—Mobile phase: ACN/H_2_O in gradient mode	DAD/FLD	Recoveries: 73–110%; RSD: NS; LOD: 2.5–10 µg/kg; LOQ: 5–150 µg/kg	[80]
15 + 1 EU priority PAHs	2 g oil in cyclohexane—after sample loading, DACC column washed with IPA, PAHs elution (backflush) with ACN (eluate concentrated and injected off-line)	Eclipse PAH C18 column, 5 cm × 4.6 mm (1.8 µm)—Mobile phase ACN/H_2_O in gradient mode	FLD/UV	Recoveries: 62.3–84.2%; RSD: 3.4–12.6%; LOD: 0.02–0.93 µg/kg; LOQ: 0.07–3.10 µg/kg	[53]
F, Pa, A, Fl, P + PAH8	According to Moret and Conte (2002) 5 g oil—SPE on 5 g silica preconditioned with 20 mL DCM, 20 mL Hex, elution with hex/DCM (8 mL, after discharging first 8 mL)—concentration and dissolution in ACN	Supelcosil LC-PAH C18 column, 250 × 3 mm (5 mm)—Mobile phase: ACN/H_2_O in gradient mode	FLD	Recoveries: 52–102%; RSD: 5–13.1%; LOQ: <0.1 µg/kg	[33]
15 of 16 EPA priority PAHs	0.4 g oil in petroleum ether—SPE on alumina preconditioned with petroleum ether, elution with petroleum ether—concentration	Hypersil Green PAH C18 column, 250 × 4.6 mm (5 μm)—Mobile phase: ACN/H_2_O in gradient mode.	FLD	Recoveries: 80–110%; RSD: 0.2–0.6%; LOD: 0.18 µg/kg; LOQ: 0.25 µg/kg	[61]
8 of the 16 EPA priority PAHs	20 g oil in *n*-Hex-M—SPE on 20 mg GOPA@Fe_3_O_4_—PAH elution with 1 mL toluene (ultrasound)—concentration and dissolution in ACN	Waters Symmetry C18 column, 250 × 4.6 mm (5 µm)—Mobile phase ACN/H_2_O in gradient mode	DAD (219 nm)	Recovery: 80.13–100.04%; RSD: 0.44–6.64% (intraday); 5.39–8.41% (interday); LOD: 0.06–0.15 µg/kg.	[81]
14 of 16 EPA priority PAHs	According to Moret and Conte (2002) with a minor modification—0.25 g oil sample instead of 2.5 g	Pinnacle II PAH C18 column 50 × 4 mm (2.1 mm)—Mobile phase: ACN/H_2_O in gradient mode	FLD	Recoveries: NS; RSD: NS; LOD: NS; LOQ: NS	[43]

ACN, acetonitrile; EtAc, ethyl acetate; CyHex, cyclohexane; DA-APPI, dopant-assisted atmospheric pressure photo ionization; DACC, donor-acceptor complex chromatography; DAD, diode array detection; DCM, dichloromethane; DMF, dimethylformamide; DMSO, dimethyl sulfoxide; FLD, fluorometric detector; GOPA@Fe_3_O_4_, phytic acid-stabilized Fe_3_O_4_-graphene oxide; GPC, gel permeation chromatography; Hex, hexane; IPA, isopropyl alcohol; PDMS, polydimethylsiloxane; MS, mass spectrometry; LLE, liquid-liquid extraction; LOD, limit of detection; LOQ, limit of quantification; MIP-SPE, molecular imprinted solid phase extraction, M-SPE, magnetic solid phase extraction; NS, not specified; RSD, residual standard deviation (repeatability); SPE, solid phase extraction; UASE, ultrasound-assisted solvent extraction; UV-Vis, ultraviolet-visible.

**Table 4:** Gas chromatography (GC) methods used for PAH determination in olive oils (2010–2020).

Target Analytes	Sample Preparation	Chromatographic Conditions	Detection	Performance Characteristics	References
16 EPA priority PAHs	5 g oil—saponification with 100 mL of 2-M KOH in H_2_O/EtOH 1:9 (*v*/*v*)—concentration and dissolution in CyHex—column chromatography on Si, elution with CyHex	5% phenyl-methylpolysiloxane capillary column 30 m × 0.25 mm (0.25 µm)—Carrier gas: helium	MS	Recoveries: ≥85%; RSD: <20% LOD: 0.1 µg/kg	[46]
PAH8	1 g oil—dSPE with mMWCNTs	5% diphenyl-95% dimethylpolysiloxane capillary column 30 m × 0.25 mm (0.25 µm)—Carrier gas: helium 30 m	MS	Recoveries: ≥87.8% to 114.4%; RSD: 1.7% to 6.2% intraday, 0.7% to 6.6% interday LOD: 0.1 to 0.88 µg/kg	[82]
16 EPA priority PAHs	1 g oil in *n*-Hex—LLE with DMF-Sonication-Back-extraction with 24 mL *n*-Hex—SPE on Florisil, preconditioned with 6 + 6 mL DCM and 6 + 6 mL *n*-Hex, elution with 5 mL of *n*-Hex/DCM 1:2, (*v*/*v*)—concentration and dissolution in ACN	5% phenyl-methylpolysiloxane capillary column 30 m × 0.25 mm (0.25 µm)—Carrier gas: helium	MS	Recoveries: 70.11–87.59%; RSD: 1.88–5.75% LOD: NS; LOQ: NS	[83]
6 of 16 EPA priority PAHs	2.0 g oil—LLE with 8 mL Hex + 8 mL DMF—SPE on 2000 mg C18, preconditioned with 20 mL MeOH + 20 mL DMF, wash with 10 mL DMF + 20 mL Milli-Q H_2_O, elution with 15 mL Hex—SPE on 2 g silica + 2 g Na_2_SO_4,_ preconditioned with 10 mL Hex, wash with 2 × 2.5 mL Hex, elution with 15 mL Hex—concentration	5% phenyl-methylpolysiloxane capillary column 30 × 0.25 mm (0.25 µm)—Carrier gas: helium	MS	Recoveries: 54.01–116.85%; RSD: 4.58–54.60% LOD: 0.04–0.48 µg/kg; LOQ: 0.12–1.34 µg/kg	[84]
16 EPA priority PAHs	0.5 g oil in CyHex-MIP—SPE (50 mg) preconditioned with 1 mL CyHex, wash with 3 mL CyHex, PAH elution with 6 mL EtAc—concentration and dissolution in toluene—(comparison with GPC and GPC + SPE)	50% phenyl polysilphenylene-siloxane capillary column 30 m × 0.25 mm (0.25 µm). 5% phenyl polysilphenylene-siloxane capillary column 1 m × 0.1 mm (0.1 µm)—Carrier gas: helium	MS (DART/TOF)	Recoveries: 70–99%; RSD: 2–11% LOD: 0.03–0.09 µg/kg; LOQ: 0.10–0.30 µg/kg	[52]
13 of 16 EPA priority PAHs	500 mg oil—LLE with 3 mL ACN-Back-extraction with 4 mL Hex-SPME (Carbopack Z/PDMS), fiber exposed for 30 min—rinse in Hex	50% phenyl polysilphenylene-siloxane capillary column 9 m × 0.10 mm (0.10 µm)—Carrier gas: helium	MS (SIM)	Recoveries: NS; RSD: 3.1–9.7% LOD: 0.03–0.25 µg/kg; LOQ: 0.10–0.83 µg/kg	[85]
15 + 1 EU priority PAHs	1.5 mL oil in *n*-Hex-LLE with 3.5 mL ACN-MIP—SPE (50 mg) preconditioned with 2.5 mL *n*-Hex, wash with 3 mL *n*-Hex + 5 mL IPA, elution with 5 mL DCM—concentration and dissolution in DMSO—dilution with H_2_O/MeOH 9:1 (*v*/*v*)	65% methyl-35% phenylsilicone capillary column 30 m × 0.25 mm (0.25 μm)—Carrier gas: helium	MS (comparison with ELISA)	Recoveries: 65–99%; RSD: NS LOD: 0.65–1.39 µg/kg; LOQ: 2.14–4.59 µg/kg	[86]
8 EPA/EU priority PAHs	20 g oil in *n*-Hex-M—SPE on 40 mg CN nanosheets, PAHs elution with 150 μL toluene	5% diphenyl-95% dimethyl polysiloxane capillary column 30 m × 0.25 mm (0.25 µm)—Carrier gas: helium	MS	Recoveries: 91.0–121.7%; RSD: intra-day 11.6%, inter-day 15.0% LOD: 0.1–0.3 µg/kg; LOQ: 0.4–0.9 µg/kg	[87]
24 PAHs in oil	0.5 mL oil in CyHex-MIP-PAH (0.5 g above) + Envi-Carb (1 g below), preconditioned with 3 mL CyHex, wash with 3 mL CyHex, elution with 8 mL toluene/EtAc 5:95 (*v*/*v*)—concentration	DB-EUPAH capillary column, 20 m × 0.18 mm (0.14 µm)—Carrier gas: helium	MS/MS (triple quadrupole)	Recoveries: 56.8–117.7% RSD: NS LOD: 0.3–0.6 µg/kg; LOQ: 0.1–2.0 µg/kg	[57]
PAH4	0.4 g of sample-SPE: C18 (bottom layer) + Florisil (upper layer) elution with ACN	DB-EUPAH capillary column 20 m × 0.18 mm (0.14 μm)—Carrier gas: helium	MS	Recoveries: 86–114% RSD: 5.2% to 7% LOD: 0.3–0.1 µg/kg	[88]
16 EPA priority PAHs + 7 EU priority PAHs	0.5 g oil—LLE with 40 ACN (saturated with Hex)—wash with 3 mL Hex—concentration and dissolution in Hex	5% phenyl-methylpolysiloxane capillary column 30 m × 0.25 mm (0.25 µm)—Carrier gas: helium	MS/MS	Recoveries: 71.5–109.9%; RSD: NS LOD: 0.1–1.0 µg/kg; LOQ: 0.3–3.3 µg/kg	[89]
16 EPA priority PAHs	1 g oil—LLE with 5 mL ACN—sonication—low temperature fat precipitation—SPE on 500 mg silica, preconditioned with 3 mL *n*-Hex, wash with 4 mL *n*-Hex, elution with 4 mL of *n*-Hex/DCM 9:1 (*v*/*v*)—concentration and dissolution in ACN	5% phenyl-methylpolysiloxane column, 60 m × 0.25 mm (0.25 μm)—Carrier gas: helium	MS	Recoveries: 84.4–113.8%; RSD: intra-day 0.9–9.2%, inter-day 1.0–10.0% LOD: 0.06–0.17 µg/kg; LOQ: 0.19–0.56 µg/kg	[90]
16 EPA priority PAHs	5 g oil in *n*-Hex-M—SPE on 3D-IL@mGO, PAHs adsorbed isolated under magnetic field, wash with 0.5 mL acetone, desorption with 0.5 mL of toluene by ultrasonic agitation	5% phenyl-methylpolysiloxane capillary column, 30 m × 0.25 mm (0.25 μm)—Carrier gas: helium	MS	Recoveries: 65.6–92.7%; RSD: 1.8–7.9% LOD: 0.10–0.60 µg/kg; LOQ: 0.33–1.98 µg/kg	[91]
16 EPA priority PAHs	0.5 g oil-LLE with 5 mL *n*-Hex + 10 mL DMF/H_2_O 9:1 (*v*/*v*)—SPE on 60 mg RP-C18, PAH elution with 350 μL ACN	5% phenyl-methylpolysiloxane capillary column, 30 m × 0.25 mm (0.25 μm)—Carrier gas: helium	MS (DSQ II quadrupole)	Recoveries: 87–104%; RSD: intra-day 4.6–5.8%, inter-day 5.3–7.1% LOD: 0.04–0.110 µg/kg; LOQ: 0.013–0.363 µg/kg	[54]
PAH4	5 g oil—LLE with 10 mL ACN/Acetone (60:40, *v*/*v*)–sonication—SPE on C18/Florisil elution with 10 mL ACN—the eluate was dried and dissolved in *n*-Hex/toluene (1 mL; 7:3, *v*/*v*)—SPE on NH2 cartridge pre-activated with *n*-Hex (12 mL), elution with *n*-Hex/toluene (10 mL; 7:3, *v*/*v*).	DB-EUPAH capillary column, 60 m × 0.25 mm (0.25 µm)—Carrier gas: helium	MS	Recoveries: 97.5–102%; RSD: 1% to 5% LOD: 0.08 to 0.01 µg/kg	[55]
13 of the 16 EPA priority PAHs	1 mL oil—LLE with 1 mL acetone + 1 mL ACN–microwave assisted—DLLME with 100 μL tetrachloroethylene	5% phenyl-methylpolysiloxane capillary column, 60 m × 0.25 mm × 0.25 µm—Carrier gas: helium	MS	Recoveries: 82.9–102.4%; RSD: <9.1% LOD: 0.2 to 2.7 µg/kg; LOQ: 0.6–9.1 µg/kg	[92]

dSPE, dispersive solid phase extraction, DLLME, dispersive liquid-liquid microextraction; mMWCNTs, multiwallet carbon nanotubes. For other abbreviation see Table 3.

## Abbreviations

The following abbreviations are used in this manuscript:

ACN	Acetonitrile
AOAC	Association of Official Analytical Chemists
APCI/APPI	Atmospheric Pressure Chemical Photoionization/Atmospheric Pressure Photoionization
APPI-MS/MS	Atmospheric Pressure Photoionization-Tandem Mass Spectrometry
BEH	Bridged Ethyl Siloxane/Silica Hybrid
BMD	Benchmark Dose
BMDL10	Benchmark Dose Lower Confidence Limit 10%
BMR	Benchmark Response
CEN	Committee of European Norms
CN	Carbon Nitride
CyHex	Cyclohexane
DAAC	Donor-Acceptor Complex Chromatography
DA-APPI	Dopant Assisted Atmospheric Pressure Photo Ionization
DAD	Diode-Array Detection
DAD/FLD	Photodiode Array/Fluorometric Detector
DART-MS	Direct Analysis in Real Time-Mass Spectrometry
DCM	Dichloromethane
DLLME	Dispersive Liquid-Liquid Microextraction
DMF	Dimethylformamide
DMSO	Dimethyl Sulfoxide
DNA	Deoxyribonucleic Acid
dSPE	Dispersive Solid Phase Extraction
EFSA	European Food Safety Authority
EI	Electron Ionisation
ELISA	Enzyme-Linked Immunosorbent Assay
EN	European Norm
EPA	Environmental Protection Agency
EtAc	Ethyl Acetate
EtOH	Ethanol
EU	European Union
EVOO	Extra Virgin Olive Oil
FAO	Food and Agriculture Organization
FDIS	Final Draft International Standard
FLD	Fluorometric Detector
FLD/DAD	Fluorometric/Diode Array Detector
FLD/UV	Fluorometric/Ultraviolet Detector
GC	Gas Chromatography
GC×GC	Comprehensive Two-Dimensional Gas Chromatography
GC-MS	Gas-Chromatography Mass-Spectrometry
GO	Graphene Oxide
GOPA@Fe_3_O_4_	Phytic Acid-Stabilized Graphene Oxide
GPC	Gel Permeation Chromatography
Hex	Hexane
HPLC	High Performance Liquid Chromatography
HPLC-FLD	High Performance Liquid Chromatography-Fluorescence Detection
IPA	Isopropyl Alcohol
ISO	international Organization For standardization
JEFCA	Joint Food and Agriculture Organization/World Health Organization Expert Committee on Food Additives
KOH	Potassium Hydroxide
LC	Liquid Chromatography
LC-LC	Coupled-Column Liquid Chromatography
LC-GC	Liquid Chromatography-Gas Chromatography
LLE	Liquid-Liquid Extraction
LOD	Limit of Detection
LOQ	Limit of Quantification
MeOH	Methanol
mMWCNTs	Multiwallet Carbon Nano Tubes
M-SPE	Magnetic Solid Phase Extraction
MIP	Molecular Imprinted Polymer
MIP-SPE	Molecular Imprinted Polymer-Solid Phase Extraction
MNP	Magnetic Nanoparticle
MoE	Margin of Exposure
MoS	Margin of Safety
MS	Mass Spectrometry
MS/MS	Tandem Mass Spectrometry
NaOH	Sodium Hydroxide
ND	Not Detected
NOAEL	No-Observed-Adverse-Effect
OO	Olive Oil
OPO	Olive Pomace Oil
PAH	Polycyclic Aromatic Hydrocarbons
PDMS	Polydimethylsiloxane
PLE	Pressurized Liquid Extraction
PTV	Temperature-Programmed Vaporization
RP	Reversed Phase
RSD	Relative Standard Deviation
SCF	Scientific Committee on Food
SEC	Size Exclusion Chromatography
SIM	Selective Ion Monitoring
SPE	Solid-Phase Extraction
SPME	Solid-Phase Microextraction
TAGs	Triglycerides
THF	Tetrahydrofuran
TS	Technical Support
UASE	Ultrasound Assisted Solid Extraction
UV	Ultraviolet
UV-Vis	Ultraviolet-Visible
VO	Virgin Olive Oil
WHO	World Health Organization
Z/PDMS	Carbopack Z/Polydimethylsiloxane
3D-IL@mGO	Three-Dimensional Ionic Liquid Functionalized Magnetic Graphene Oxide Nanocomposite
